# Development of a High-Sensitivity Electrochemical Immunoassay Using a Fully 3D-Printed Electrocatalytic Microelectrode Probe Platform

**DOI:** 10.1021/acs.analchem.5c08037

**Published:** 2026-03-19

**Authors:** Niamh Docherty, Chloe L. Miller, Alexandra Dobrea, Daniel Macdonald, Alisdair Gordon, Susan Pang, Ying Fu, Melanie Jimenez, Damion K. Corrigan, Bhavik Anil Patel

**Affiliations:** † University of Strathclyde, Thomas Graham Building Centre for Advanced Measurement Science and Health Translation, Pure and Applied Chemistry, 295 Cathedral St, Glasgow G1 1XL, U.K.; ‡ School of Applied Sciences, Centre for Lifelong Health, University of Brighton, Brighton, East Sussex BN2 4GJ, U.K.; § 3527 University of Strathclyde, Biomedical Engineering, Wolfson Centre, 106 Rottenrow East, G4 0NW Glasgow, U.K.; ∥ National Measurement Laboratory at LGC The Priestley Building, Guildford, Surrey GU2 7XY, U.K.; ⊥ 3527 University of Strathclyde, Pure and Applied Chemistry, Technology Innovation Centre, 99 George Street, Glasgow G1 1RD, U.K.

## Abstract

Electrochemical biosensors are a promising route to point-of-care diagnostics, yet their translation is hindered by the need for electrode surface functionalization, which introduces variability, increases cost and production complexity, and limits scalability and stability. Additive manufacturing using conductive filaments for rapid fabrication of three-dimensional (3D) electrodes overcomes these limitations. This study evaluates composite filaments comprising polylactic acid (PLA), carbon black (CB), and multiwalled carbon nanotubes (MWCNTs) for fused filament fabrication (FFF) of electrochemical electrodes for indirect detection via TMB^+^ measurement. Both the filament composition and electrode size were directly compared to determine the most suitable electrode architecture for sensitive measurements in complex samples. As a key novelty, the electrode diameter was reduced from 1 mm to the microscale to investigate the influence of electrode size on electron transfer efficiency. To improve measurement consistency and throughput, a 3D-printed assay accessory (“The Consistent Dipper”) was developed to guide electrode immersion and reduce movement during amperometric measurements. PLA/MWCNT microelectrodes exhibited increased current density and reduced background noise compared to carbon black filament electrodes. The MWCNT microelectrodes were subsequently applied to a cardiac troponin I (cTnI) electrochemical immunoassay and in undiluted human serum, a cTnI detection limit of 7.4 pg mL^–1^ was achieved, representing an approximately 19-fold improvement compared to PLA/MWCNT macroelectrodes (140 pg mL^–1^). Following optimization to reduce incubation times, a clinically relevant detection limit of 85 pg mL^–1^ was obtained within a total assay time of 1 h. By combining enhanced electrochemical performance with low-cost, flexible FFF-printed microelectrodes, this platform provides a scalable route to rapid immunodiagnostics. This study represents the first application of microscale 3D-printed PLA/MWCNT electrodes for clinical biomarker detection using a readily manufacturable sensor system.

## Graphical abstract

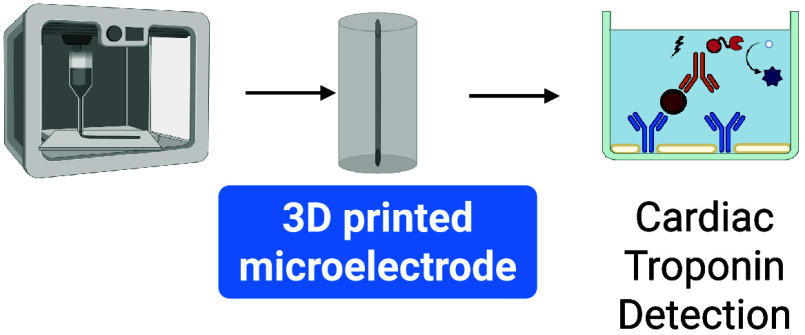

Cardiac troponin is the gold standard test for acute myocardial infarction diagnostics. Despite the use of other tests such as electrocardiograms, a cardiac troponin blood test is required to rule in or out a heart attack in high-risk chest pain patients.^1,2^ The troponin complex comprises three subunits (I, C, and T), with troponins I (cTnI) and T (cTnT) most commonly measured in suspected heart attack patients. Current diagnostic pathways rely on laboratory-based immunoassays.^3^ These established techniques have excellent sensitivity and specificity but require sample transport, dedicated equipment, trained personnel, and have a ‘time to result’ of several hours. The reliance on cTnI tests for AMI diagnosis places immense strain on emergency healthcare resources, slowing discharge of noncardiac patients and delaying urgent cardiac care. The situation is exacerbated as a small proportion of chest pain is heart attack-related, and the volume of chest pain complaints is rising every year.^4^ Therefore, a faster cTnI test to identify AMI patients would save time, money, and lives.

Point-of-care testing (POCT) has therefore become a major research priority for heart attack diagnostics with the goal of enabling earlier triage to exclude low-risk patients. A key challenge is the extremely low physiological concentrations in healthy individuals of below ∼15 pg mL^–1^, meaning high sensitivity is needed to detect early AMIs.^5^ In recent years, POC cTnI platforms have been released.^6–8^ However, the uptake of these benchtop devices is limited due to roadblocks related to cost, operator requirements, sample handling, and benchtop device requirements.^6^

Electrochemical immunosensors combine the inherent high analytical sensitivity and target specificity of antibodies with the rapid response and miniaturization potential of electrochemical detection.^9,10^ While other recognition elements such as molecularly imprinted polymers (MIPs) and aptamers have merit, their adoption in real-world diagnostics remains limited. For cTnI particularly, antibodies persist as the gold-standard bioreceptors because they provide clinically validated affinity, well-defined epitope recognition, and established manufacturing routes that meet regulatory requirements. As such, commercially available cTnI platforms rely on antibody-based detection, despite advances in alternative biorecognition chemistries. A common redox reporter system for electrochemical immunosensors is the horseradish peroxidase (HRP) enzyme-based system, characterized by its participation in redox reactions with its substrates.^11,12^ Figure 1A presents the reaction schematic for 3,3′,5,5′-Tetramethylbenzidine (TMB), a common HRP substrate. HRP catalyzes the oxidation of TMB, yielding two distinct colored products. The one-electron oxidation produces a blue charge-transfer complex (TMB^+^). Further oxidation facilitated by a strong acid such as H_2_SO_4_, produces yellow diamine known as TMB^2+^.^13^ TMB ^2+^ is stable and widely used in colorimetric assays as a reporter molecule, whereas TMB^+^ is used as a electroactive redox mediator. Together, the choice of bioreceptor and labeling system plays a crucial role in the biosensor performance.

**Figure 1:**
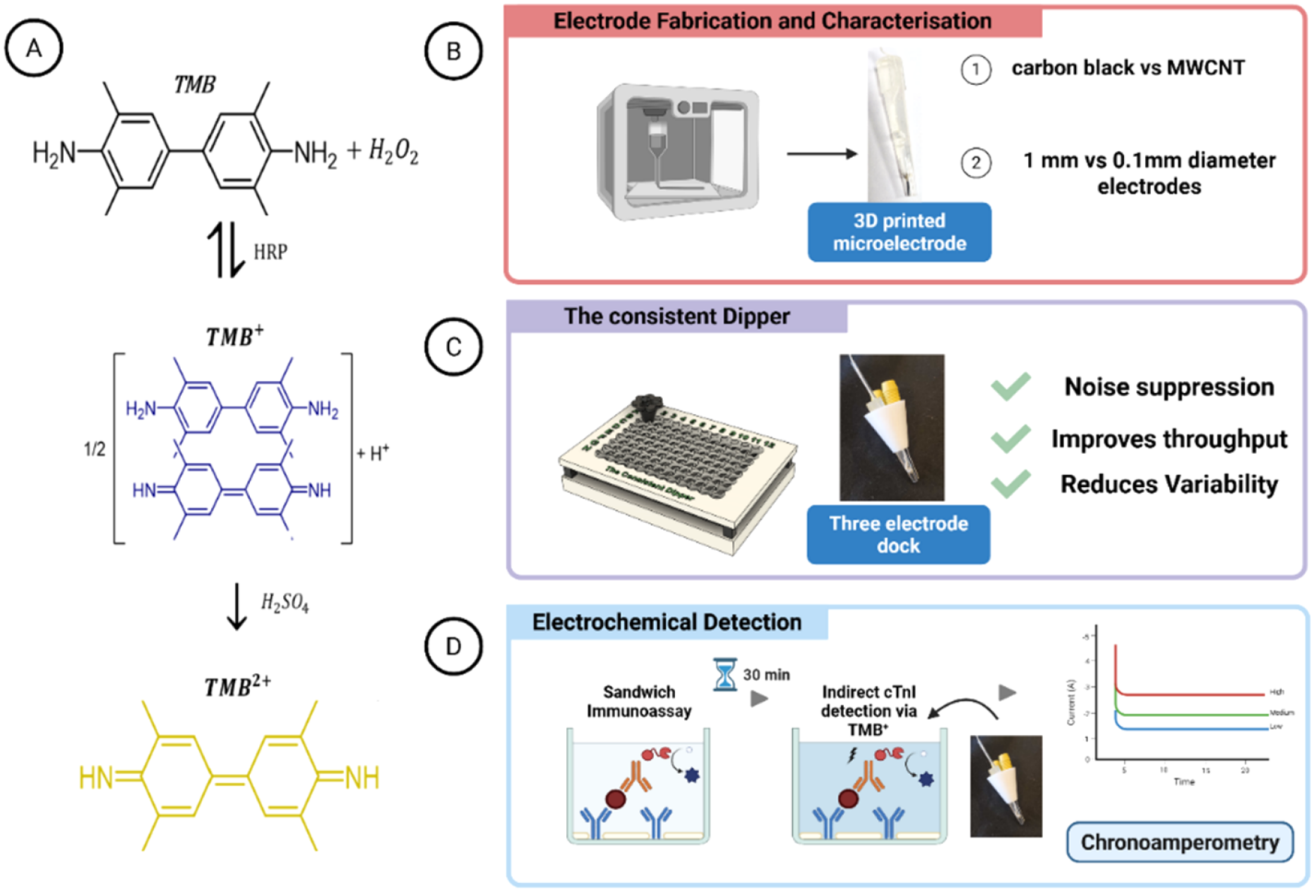
Schematic overview of 3D-printed miniature electrodes and off-electrode detection of cardiac troponin I. (A) Reaction schematic of the oxidation of 3,3′,5,5′-Tetramethylbenzidine (TMB). In the presence of hydrogen peroxide (H_2_O_2_) and horseradish peroxidase (HRP), TMB undergoes a one-electron oxidation to form the blue-colored radical cation (TMB^+^). Upon the addition of an acidic stop solution, a second oxidation occurs, converting TMB^+^ to the yellow diamine form (TMB^2+^). (B) Electrode manufacturing and screening to determine the optimal filament and electrode diameter. (C) Custom 3D-printed accessory designed to help move electrodes between samples and hold the electrodes in place to suppress noise. (D) Final electrochemical assay with chronoamperometric readout from the reduction of TMB^+^ at the MWCNT/PLA electrode surface. Together, these panels summarise the key components of the sensing platform and assay workflow described in this study. Figure created in Biorender.

The sensitivity of electrochemical immunosensors is predominantly dictated by the antibody–antigen interaction, which requires an unobstructed antigen-binding region to capture the analyte target.^14^ Thus, antibody orientation is critical to maximize the number of available binding sites. Researchers have developed various immobilization methods that aid in controlling the orientation and distribution of antibodies and bring the target/reporter closer to the electrode surface. These techniques include physical adsorption, carbodiimide (EDC) in combination with *N*-hydroxy succinimide (NHS), thiol–gold binding, and the use of polypyrrole films.^15^ Comparative studies have shown that the choice of immobilization method affects immunosensor performance.^16^ This agreed with previous work that demonstrated more controlled immobilization strategies yielded superior limit of detection (LOD).^7^ The study compared off-electrode target capture and transferring the solution to the electrode with physisorption and chemisorption, each achieving varied detection limits for cTnI in diluted serum (1000 pg mL^–1^, 130 pg mL^–1^, and 109 pg mL^–1^, respectively).^7^

Since our understanding of electrode surface interactions has grown, surface functionalization has emerged as a significant bottleneck in electrochemical sensor development. In particular, antibody orientation cannot be fully controlled, leading to uneven receptor loading or masking of antigen-binding regions, which increases assay variability and reduces stability.^15^ In addition, immobilized layers can impede electron transfer, often necessitating signal-amplification approaches such as nanomaterials or conductive coatings to restore performance.^17^ Furthermore, consistent electrode functionalization is challenging to reproduce on a larger scale. These limitations impede electrochemical immunosensors translation to real-world use, motivating interest in sensitive detection platforms that operate without surface functionalization. Advances in miniaturization, automation, and system integration have further encouraged the re-evaluation of simpler measurement approaches for improved translatability.^14^

Another crucial electrochemical biosensor component is the electrode itself. A range of electrode architectures is commonly employed, including laser-ablated, interdigitated, and screen-printed electrodes, each with distinct advantages and limitations. Laser-ablated gold electrodes deliver high precision and reproducibility but rely on expensive materials and specialized equipment. While they are chemically stable and easily regenerated, electrode geometry and surface tunability are limited and restricts functionalization options.^18^ Interdigitated electrodes (IDEs) offer excellent sensitivity and well-established microfabrication routes. However, IDE fabrication often requires a cleanroom, which is impractical for low-resource or rapid-development settings. Field-effect transistors (FETs) have numerous well-documented advantages, yet are strongly susceptible to surface fouling.^19^ These constraints have driven interest in alternative electrode manufacturing approaches that eliminate surface functionalization and enable sensitive, scalable electrochemical assays.^20,21^

3D-printed electrochemical sensors have gained significant popularity in recent years, due to their unique advantages, such as rapid iteration production, customization of materials, and the ability to fabricate diverse geometries.^21^ Fused filament fabrication (FFF) stands out as a preferred method for producing electrochemical sensors because it is economically and environmentally friendly, biocompatible, and does not degrade into toxic byproducts.^21–23^ To make electrochemical sensors, carbon allotropes, such as carbon black and multiwalled carbon nanotubes (MWCNT), are mixed with polylactic acid (PLA).^24,25^ A wide range of conductive filaments for FFF printing exists, with customized filament gaining traction as it enables a higher carbon load,^26^ added flexibility through plasticizers,^27,28^ and the opportunity to add nanoparticles to the filament.^29,30^

3D printing offers the unique capability to create electrodes in both micro and macro sizes,^31,32^ design surface geometries to increase surface area,^17,29^ and shape electrodes to mimic biological structures.^33,34^ Microelectrodes are advantageous because they facilitate enhanced mass transport to the electrode and produce less background noise.^35–37^ While 3D-printed electrodes (3DPEs) are used for environmental monitoring and small-molecule detection, their application to clinically relevant biomarker sensing remains comparatively underexplored.^38^ In particular, translation to complex biological matrices and low-abundance protein targets such as cardiac troponin presents additional challenges that have yet to be systematically addressed. Recent studies have explored the use of PLA/MWCNT composites for small-molecule detection such as direct serotonin detection^31^ and glucose and dopamine using 3D-printed electrodes.^39^ With continued improvements in conductive filaments and smaller feature sizes, 3D printing could feasibly support the development of sensitive, assay-ready electrode architectures. Overall, while laser-ablated IDE and FET-based platforms set a benchmark for reproducibility and signal performance, FFF remains a more flexible and economical method for prototyping and adapting sensor architectures to specific assay needs.

Against this backdrop, this study investigates the application of 3D-printed electrodes composed of a PLA/MWCNT composite material for cTnI detection. To our knowledge, this is the first investigation of 3D-printed FFF PLA/MWCNT for cTnI detection. This study fabricated and characterized multiple 3D-printed carbon electrode designs to validate the use of an MWCNT-based filament and reduced electrode dimensions to substantially improve performance by promoting microelectrode-like behavior. The identified optimal electrode design was then used for cTnI detection. To minimize variability associated with manual handling, a custom movement accessory (“The Consistent Dipper”) was designed to diminish noise and improve reproducibility. The final stage of the study demonstrated the detection of cTnI in undiluted serum and explored opportunities to further streamline and shorten the assay protocol (see overall workflow in Figure 1).

## Experimental Section

### Reagents

Details of all reagents used are listed in Supporting Information 1

### Electrochemical Detection System

#### 3D-Printed Electrode Production

Polylactic acid/multiwalled carbon nanotube (PLA/MWCNT) filament (3DX Tech, USA) was used for FFF electrode fabrication. A 10 cm^3^ cube design was modeled in SolidWorks and printed on a FlashForge Creator Pro 2 printer. The print was paused halfway through when the outer perimeter was formed, creating strands for electrode manufacture. The extruder temperature was 230 °C, and the bed temperature was maintained at 50 °C. Two print layer thicknesses were used (0.1 and 1 mm) to create a macro- and microsized version for comparison. Each strand was cut to a length of 5 mm, and an ohmic connection was made by attaching a silver wire using silver epoxy resin. The electrode was then placed in a pipet tip, sealed with epoxy resin, and left to set for 48 h. To expose the 3DPE surface, a diamond saw was used to remove the tip end. Finally, the 3DPEs were polished using sandpaper (400 and 200 grit). The process was repeated for the carbon black/polylactic acid (PLA/CB) filament. Further details can be found in the methods and Supporting Information of Xue et al.^31^

#### The Consistent Dipper Accessory

The Dipper consists of a base with four standoffs, a docking guide with 96 docking holes aligned with the 96 wells of a microplate, and a three-electrode dock (TED) (Figure 3A). The microplate was placed in the base, and the docking guide supported by the four standoffs was positioned above the microplate. The TED has three slots, one for each electrode, thereby forming an easy-to-handle electrode cell. In addition, when placed into the docking guide, a 30° rotation inserted a protruding pin at the base of the TED into a groove in the docking hole (Figure 3B), ensuring the electrode remained steady, was aligned with each well, and inserted to the same depth each time the EC signal was measured. The Ag/AgCl reference and Pt wire counter electrodes were soldered to 2 mm banana sockets (RS Stock No.: 280–8773) for secure, straightforward connection to the potentiostat leads. The 3DPEs were installed to the slot custom-built for the pipet tip shape of the electrodes (*cf*. Supporting Information 2). While the counter and reference electrodes were glued in for secure attachment, the working electrode was inserted and held in place via an interference fit, facilitating easy installation and removal of the electrode and minimizing play during the measurement process. Finally, to facilitate gripping during use, the top rim of the TED was designed to resemble a thumb screw (Figure 3B). All parts of the Dipper were designed in the Fusion 360 software before being sliced in the Bambu Studio app and 3D-printed using a Bambu Lab A1 3D printer using standard PLA filament. The print files for the Dipper are available at https://github.com/ADobrea797/The-Consistent-Dipper, and an example of its use during electrode testing can be seen in Supporting Video S1.

### Physical Characterization

#### Field-Emission Scanning Electron Microscopy (FE-SEM)

FE-SEM images were obtained using a Zeiss SIGMA field-emission gun SEM equipped with an Everhart–Thornley detector operating in secondary electron detection mode, using a 5 kV accelerating voltage, a 20 μm aperture, and an 8.1 mm working distance.

#### Raman Spectroscopy

Raman spectra were acquired using a (laser wavelength, 532 nm) excitation source with a 20× objective lens and an integration time of 5 s. The laser power at the sample was maintained at 100% (max laser power output 500 mW). Spectra were collected over the 100–3500 cm^–1^ range. Sample spectra were recorded at three different positions to account for surface heterogeneity, and the spectrum shown is the average of three spectra with no preprocessing. A white light image was taken by using a 5× objective.

### Electrochemical Measurement

#### Electrochemical Properties of 3,3′,5,5′-Tetramethylbenzidine When Measured on Using 3DPEs

25 ng of HRP B (R&D Systems) was added to the TMB substrate solution (R&D Systems), and after 1 min, the electrochemical analysis was performed. Cyclic voltammetry (CV) was then performed under the following conditions: −0.5 to 0.5 V range, 100 mV/s scan rate, using a three-electrode system of a working electrode, a bleached silver wire as the reference electrode, and a platinum counter electrode. Next, the HRP and TMB substrate mixture was prepared again, and after 1 min, the stop solution (2 M H_2_SO_4_) was then added prior to CV under the same conditions.

#### Micro-MWCNT Electrode Characterization

CV was performed at −0.7 to 0.2 V for various concentrations of Hexamineruthenium­(II) chloride (RuHex) in 0.1 M KCl (2, 5, and 10 mM). For baseline comparison, 0.1 M KCl in 1× PBS was tested under equivalent CV conditions.

#### Pretreatment Study

Electrodes were immersed in 0.5 M NaOH prepared in 1× PBS and subjected to chronoamperometry at +1.4 V for 200 s, followed by −1.0 V for 200 s and rinsed thoroughly with 1x PBS prior to use. To assess pretreatment effects on TMB^+^ detection, electrodes were sanded and characterized by CV in the TMB substrate. After activation, CV was repeated using a 100 mV/s scan rate over −1.0 to 1.0 V.

#### Regeneration Study

Used electrodes were stored in a cool, dry container for 1 week. CV at 100 mV/s with 1 mM RuHex was performed to establish the baseline electrochemical behavior after use. The electrodes were sanded with 800-grit sandpaper, and CV was repeated.

#### Electrode Area Calculation

Electrode areas were estimated from the CV of 1 mM Ruhex based on the simplified room temperature form of the Randles–Sevcik eq (eq 1).

1 $$i_{p}=2.69\times 10^{5}n^{3/2}{\mathrm{AD}}^{1/2}{\mathrm{Cv}}^{1/2}$$

In eq 1, *i*_p_ is the peak current (A), *n* is the number of electrons transferred, *A* is the electroactive surface area (cm^2^), *D* is the diffusion coefficient of the redox species (cm^2^s^–1^) (8.4 × 10^–6^ cm^2^/s for Ruhex),^40^ *A* is surface area of the working electrode (cm^2^), *C* is the concentration of the redox species (mol cm^–3^), and *v* is the scan rate (V s^–1^) (0.1 V/s).

#### cTnI Immunoassay

cTnl immunoassay was developed and carried out in a 96-well microtiter plate for both absorbance and electrochemical assay formats. Each well was coated with 2 μg of the monoclonal capture antibody (19c7) and incubated overnight at room temperature. The wells were washed three times with 0.05% Tween-20 in 1× PBS, discarding the contents and removing residual liquid. To minimize nonspecific binding, 1% bovine serum albumin (BSA) in PBS was added as a blocking agent to each well and incubated for 1 h at room temperature, followed by the same washing procedure. Undiluted cTnI depleted human serum was centrifuged at 5000*g* for 10 min and the lipid layer was removed. A standard curve was prepared by serial dilution of cardiac troponin IC (cTnIC) with the prepared serum either undiluted (100%) or diluted to 10% in 1× PBS. Standards and samples were added to the wells and incubated for 2 h at room temperature. To remove excess sample, wells were washed three times before adding 400 ng of the monoclonal detection antibody conjugated to HRP (42c-HRP) and incubated in the dark at room temperature for 2 h. The wash step was repeated before adding the TMB substrate solution. The plate was incubated in the dark at room temperature for 30 min. For absorbance measurements, the reaction was stopped by adding stop solution (2 M H_2_SO_4_), producing the TMB^2+^ complex (yellow), and absorbance was measured at 450 nm using a microplate reader.

#### Electrochemical Immunoassay

For electrochemical readout, a 3DP working electrode, a bleached silver wire (reference electrode), and a platinum wire (counter electrode) were inserted into each well, and chronoamperometry was performed at −0.05 V for 15 s to record the current response. Electrodes were rinsed with 1× PBS between measurements and reused for up to 10 consecutive measurements before being replaced

#### Reproducibility Study

The electrochemical immunoassay was repeated on three separate occasions. Reproducibility was evaluated using three sample types: nonspiked cTnI-depleted human serum (sample blank) and spiked with 25 and 50 pg cTnIC per mL of serum. Each sample type was done in replicate (*n* = 4), and samples were analyzed using four electrodes. Each electrode was used a total of three times: to analyze one nonspiked and both spike level samples. All electrodes were rinsed thoroughly with 1× PBS before reuse.

#### Specificity Study

Assay specificity was assessed by analyzing six replicates of nonspiked and spiked (100 pg mL^–1^) cTnI-depleted human serum using a set of six electrodes. Each electrode was used to analyze one nonspiked and one spiked sample, and all electrodes were rinsed with 1× PBS before reuse. All measurements followed the standard 2 h sample and detection antibody incubations protocol.

#### Accelerated Immunoassay

The immunoassay procedure was repeated using shorter sample and detection antibody incubation times. A screening was performed using spiked 10% human serum samples to determine whether heating and mixing retained sensitivity while reducing incubation times. Additionally, the primary antibody concentration was doubled from 2 to 4 μg. After screening, optimal conditions were identified the optimized accelerated immunoassay protocol was repeated with undiluted spiked cTnI-depleted serum.

#### Data Analysis

The mean current at 15 s (electrochemical) or mean absorbance (optical) was calculated for each measurement to generate cTnI dose–response curves with concentration (*x*-axis) plotted on a semilogarithmic scale. A 4-parameter logistics fit was then fitted onto the mean signal response value for each concentration. The LOD was calculated from the blank signal using the IUPAC definition (LOD = *S̅*_blank_ + 3σ_blank_), which produces a *y*-value representing the detection threshold. This *y*-value was substituted into the respective 4PL fit equation to determine the corresponding cTnI concentration.

To determine the impact of Consistent Dipper, electrochemical immunoassay data generated with and without the use of Consistent Dipper were collated for comparison. Samples of equivalent cTnI concentration were selected, and data were based on the treatment or condition applied. For each sample, the mean current (*I̅*_*i*_) from 3 to 15 s was calculated using eq 2.

2 $${\mathrm{I̅}}_{i}=\frac{1}{n}\Sigma_{t=1}^{n}I_{i,t}$$

Where *I*_*i*,*t*_ = current at time point *t* for sample *i*, and n was the number of valid points in sample *i*. The root-mean-square error (RMSE) was used to quantify the amount that each valid data point deviates from the mean signal over time for each sample (eq 3).

3 $$\mathrm{RMSE}=\sqrt{\frac{1}{n}\Sigma_{t=1}^{n}{\left(I_{i,t}-{\mathrm{I̅}}_{i}\right)}^{2}}$$

*I_i,t_* is the current at time point t for the sample, *I̅*_*i*_ the mean current for the sample, and *n* the number of valid time points as above. The steady-state current, RMSE, and summary statistics, including the mean and standard deviation of RMSE, were computed for both groups in MATLAB and the data exported to OriginPro for visualization. To aid the comparison of different experimental conditions and electrodes, the signal-to-noise ratio (SNR) was calculated using eq 4.

4 $$\mathrm{SNR}=\frac{S-B}{\sigma_{B}}$$

Where *S* is the mean signal of the lowest standard in the dose response curve, B is the mean signal of the negative control, and σ_B_ is the standard deviation of the negative control.

## Results and Discussion

### MWCNT Enhancement and Reducing Electrode Dimensions Improves Sensitivity

Electrodes were fabricated from 3D-printed polylactic acid/carbon black (PLA/CB) and polylactic acid/multiwalled carbon nanotube (PLA/MWCNT) filaments in two diameters: macroelectrodes (1 mm) and microelectrodes (100 μm), yielding four electrode types (macro-CB, macro-MWCNT, micro-CB, and micro-MWCNT). The electrodes were compared to assess the effect of the filament material and electrode size on TMB detection. Each electrode was immersed in a TMB^+^ solution and a TMB^2+^ solution to determine the optimal electrode and conditions for cTnI detection further downstream.

Figure S1.1 presents the raw cyclic voltammograms for the four electrode configurations. In general, MWCNT-based electrodes exhibited higher currents for TMB^+^ than for TMB^2+^. For example, the micro-MWCNT electrode produced the largest response to the blue TMB^+^ solution, with a reduction peak of −1.21 nA, compared to −0.451 nA for the acidified yellow TMB^2+^ solution. The reduced current observed for TMB^2+^ arises from the largely irreversible nature of the second oxidation step, which limits redox cycling and results in smaller peak currents compared to the quasi-reversible TMB^+^ system. Consequently, the blue TMB^+^ solution was used for subsequent comparisons of the electrode size and filament material.

For a fair comparison between electrode sizes, current density was calculated using the filament thickness as an estimate of the electrode area. Measured currents were ∼50 nA for macro-CB and ∼1 nA for micro-CB. In contrast, MWCNT-containing electrodes showed substantially higher signals (∼400 nA for macro-MWCNT and ∼100 nA for micro-MWCNT), reflecting the enhanced conductivity and surface area of the MWCNT/PLA filament. Figure 2A shows that both macro-CB and micro-CB exhibited high solution resistance and large capacitive currents. When normalized to electrode area, the micro-MWCNT electrode displayed a significantly higher cathodic peak current density (∼1.51 μA/m^2^) than the macro-MWCNT, with well-defined peaks, lower background noise, reduced capacitance, and improved mass transport. Based on these findings, subsequent experiments utilized micro-MWCNT electrodes for the detection of TMB^+^ in indirect cTnI detection.

**Figure 2:**
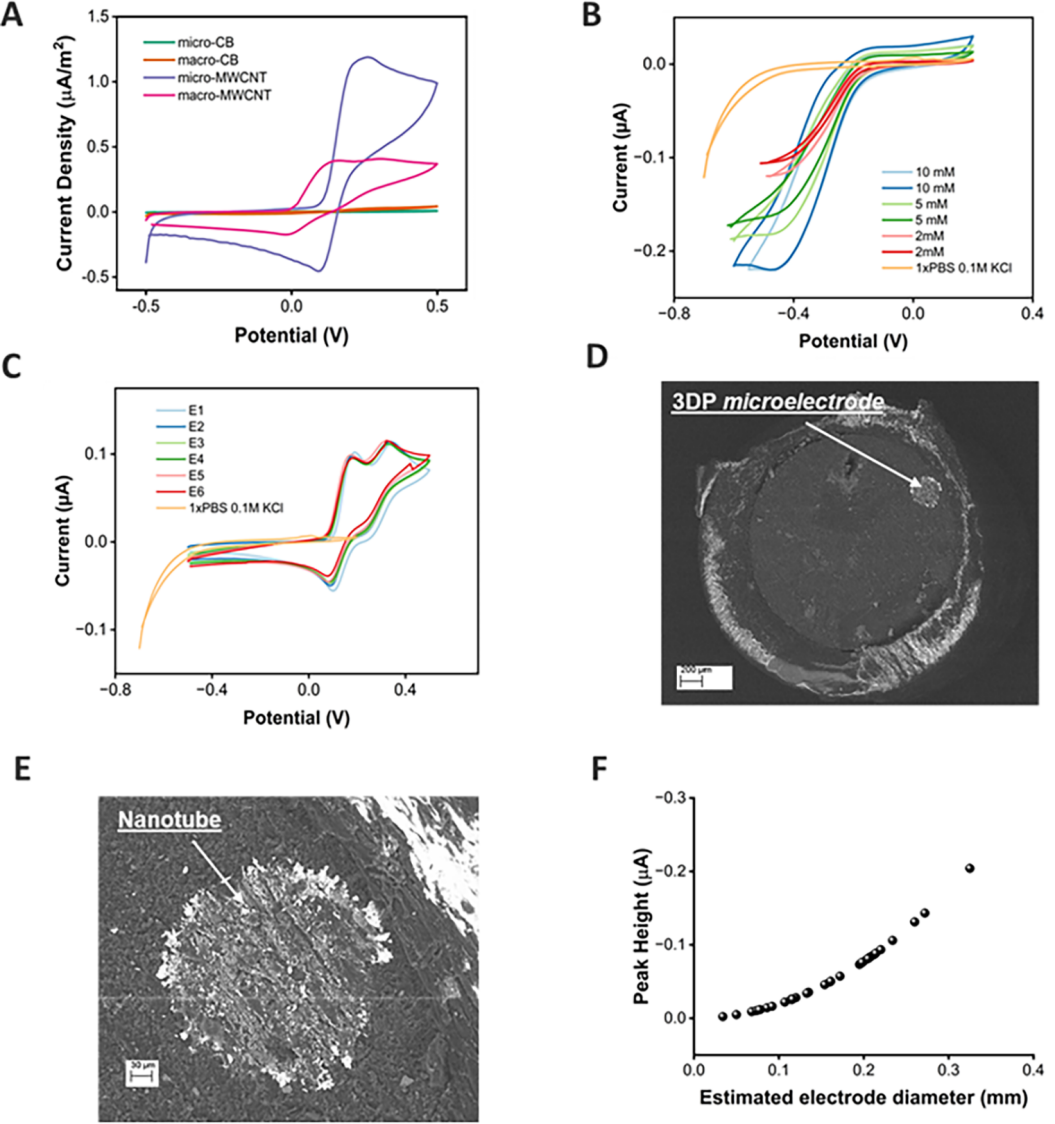
Electrode characterization. (A) Current density of macro-CB, micro-CB, macro-CNT, and micro-MWCNT electrodes in TMB substrate-HRP solution with a shared platinum reference/counter electrode. (B) Cyclic voltammetry of 5 mM RuHex for the micro-MWCNT electrodes (platinum reference/counter). (C) Cyclic voltammetry of TMB substrate solution, with the micro-MWCNTs, an Ag/AgCl reference, and a platinum wire counter electrode. (D) SEM analysis of micro-MWCNT electrode showing the 3DPE within the epoxy casing. (E) SEM of micro-MWCNT electrode showing clusters of nanotubes and (F) cluster plot of anodic peak height for RuHex screening of electrodes against estimated electrode diameter (*n* = 35).

After selecting the micro-MWCNTs for electrochemical immunoassay use, the micro-MWCNTs were characterized with RuHex to understand the general electroanalytical performance of the electrodes. In Figure 2B, the micro-MWCNT electrode exhibits microelectrode-like behavior with enhanced mass transport, demonstrated by the current plateau due to the consistent response of fresh electroactive species due to radial diffusion. The absolute current response reduced with RuHex concentrations due to reduced availability of the electroactive species. Despite having a geometric diameter of 0.1 mm (100 μm), exceeding the typical microelectrode diameter (1–50 μm), the electrodes displayed steady-state Ru­(NH_3_)_6_^3+^/Ru­(NH_3_)_6_^2+^ responses consistent with microelectrode behavior and are therefore described as microelectrodes in the context of this study. Figure 2C shows the capacity of micro-MWCNT to resolve subtle electrochemical features compared to the other electrode types (Figure S1.1), such as the reduction of TMB^+^ at about 0.1 V. Cyclic voltammetry of the micro-MWCNT electrodes in buffer (1× PBS, 0.1 M KCl) shows the low capacitance of the electrodes, leading to minimal background signal. Scanning electron microscopy images of the micro-MWCNT electrodes show the 3DPE encapsulated in epoxy (Figure 2D) and the presence of nanotubes upon the surface of the electrode (Figure 2E). Raman spectroscopy available in the Supporting Information confirmed the characteristic D (∼1350 cm^–1^) and G (∼1600 cm^–1^) bands of micro-MWCNTs, though peak definition was limited (Figure S1.2). The moderate signal quality likely arose from the thin curved wire geometry, MWCNT structure, and local laser-induced heating, all of which reduced the uniform coupling and broadened the vibrational features.

Before conducting broader experiments, electrode pretreatment was trialled to optimize the electrode surface before use. Although previous studies using similar electrode materials reported that alkaline pretreatment improved electrochemical behavior.^31,32,41^ This was not observed here. Figure S1.3 (Supporting Information) shows that untreated electrodes produced lower baseline noise and well-defined redox peaks compared to those after NaOH multistep amperometry treatment. Since the electrode performance was satisfactory, no further pretreatment was applied after polishing. Stability studies indicate that once opened, air exposure degrades the PLA/MWCNT exposed surface (Figure S1.4). However, due to the 3D dimensions and flexibility of the materials used, the electrode current exhibition was restored by polishing the surface to reveal a fresh surface on the 3DPE and reused (Figure S1.4).

To verify consistent electrochemical behavior across the batch, micro-MWCNT electrodes were assessed using CV in 0.5 mM RuHex solution. Figure 2F shows the distribution of the electrode peak height and estimated diameter for fresh, unused, polished micro-MWCNT electrodes. Anodic peak currents ranged from −0.002 to −0.204 μA, corresponding to estimated electrode diameters of ∼0.034–0.325 mm. The mean electrode diameter was 0.168 ± 0.073 mm (CoV 43.5%). Most electrodes exhibited Δ*E*_p_ values between 0.11 and 0.14 V, indicating broadly similar electron transfer kinetics across the array. Such variability is expected with 3DPEs, as the 0.1 mm printing resolution cannot perfectly maintain dimensional tolerances. To address this, all electrodes were screened prior to immunoassay use, and only those within ±15% of the mean estimated diameter were selected to ensure reproducible results in the cTnI biosensing experiments.

Overall, incorporating MWCNTs into the PLA matrix markedly enhanced the TMB^+^ interaction, catalyzing electron transfer and significantly improving the sensitivity of the sensing platform. Reducing the electrode diameter from 1 mm to 0.1 mm increased the current density and improved signal responsiveness, likely due to a higher electroactive surface-area-to-volume ratio and reduced resistance along the conductive pathway.

### The “Consistent Dipper” Accessory Reduces Noise and Data Collection Time

After demonstration of the electrochemical performance of the micro-MWCNT electrodes, a practical limitation became clear when running amperometric measurements with larger sample sizes. Individually clipping and transferring electrodes between wells was slow, labor-intensive, and introduced handling variability (Figure S1.5). This was especially problematic for immunoassays, where the enzymatic reaction is time-dependent and consistent timing is critical. To address the timing issue and reduce the dexterity required for low-noise measurements, a 3D-printed three-electrode docking system, the “Consistent Dipper,” was developed. Figure 3A shows the accessory design, which incorporates fixed reference and counter electrodes and accommodates a three-electrode configuration within a single well. The rig functions as a docking system for the plate, precisely guiding the electrode cell contained in the TED into each well. The Consistent Dipper shaded the samples from light during measurements, minimizing the light effect on the enzymatic reaction. The TED is secured in place via a groove interaction between the TED and the docking guide, ensuring the user places the electrodes into each sample in a consistent manner and minimizes noise from unintentional electrode movement.

**Figure 3:**
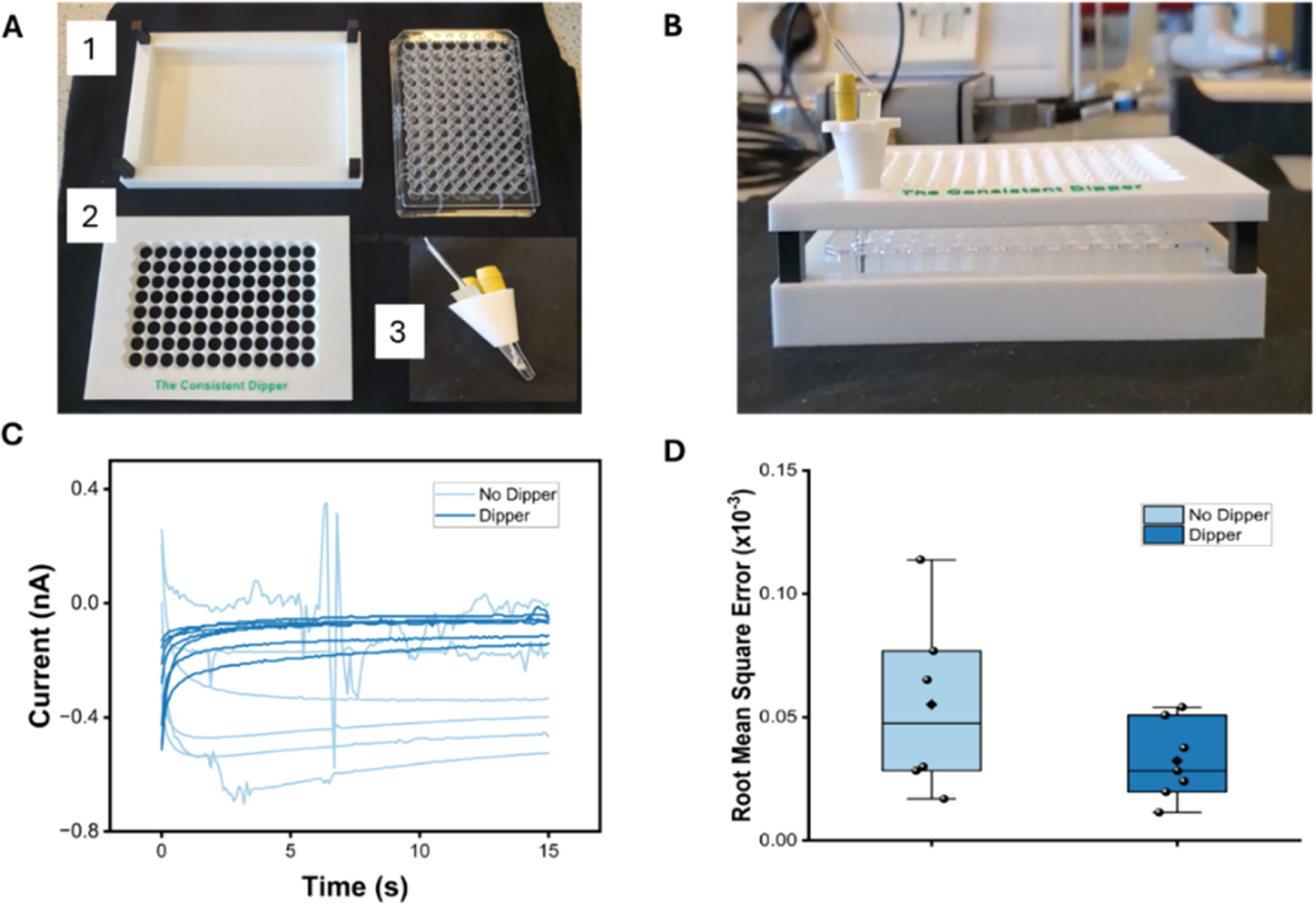
The Consistent Dipper: (A) Exploded view showing the components. (1) Plate support and spacers (2), the docking guide (3), the three-electrode dock (TED). A 96-well plate sits on a support base with spacers to secure the TED. The docking guide features 96 holes and a ratchet mechanism that ensures consistent electrode immersion depths in each well. The working electrode can be exchanged, while the reference and counter electrodes remain fixed. (B) Fully assembled system positioned over a 96-well plate in operational mode. (C) Chronoamperometric response comparison of 50 pg mL^–1^ cTnI samples with and without the Dipper accessory. (D) Boxplot of the root-mean-square error calculated for the samples with and without dipper (*n* = 6).

Figure 3C compares chronoamperometric measurements acquired with and without the Consistent Dipper. In the absence of the accessory, uncontrolled electrode movement during measurement led to signal disruption and high variability between identical samples. Incorporation of the Consistent Dipper stabilized the electrode, reducing noise and improving signal consistency. To quantify variability between measurements, RMSE was calculated for replicates performed with and without the dipper accessory. RMSE reflects the deviation from the mean current during each measurement, allowing for a direct comparison between the configurations. Figure 3D shows the reduction in variability following the applied condition. The No-Dipper group exhibited a mean RMSE of 5.51 × 10^–5^ with a standard deviation of 3.70 × 10^–5^, whereas the Dipper group showed a lower mean RMSE of 3.22 × 10^–5^ with a standard deviation of 1.60 × 10^–5^, indicating the dipper led to more consistent current measurements across samples. Importantly, the accessory significantly reduced data acquisition time from ∼1 min to ∼5 s between measurements ensuring each sample undergoes a similar TMB incubation period before the signal measurement. This simple yet effective accessory represents an innovative modular support that improves user friendliness and addresses a common source of measurement noise, thereby enhancing the overall signal reproducibility.

### Electrochemical-Based Detection of cTnI with Micro-MWCNT Electrodes

Following electrode characterization, a conventional optical assay was used to validate antibody pair selection and generate a standard curve using diluted spiked serum samples (Figure S1.6). The response exhibited a lower plateau at minimal concentrations, followed by a sigmoidal increase at higher analyte levels due to limited TMB oxidation at low concentrations. Despite this plateau, concentration differences remained distinguishable, achieving an LOD of <12.5 p pg mL^–1^, demonstrating a very low background signal and confirming effective antigen–antibody binding and providing a reference for electrochemical performance.

After validation of the antibodies and immunocomplex formation, chronoamperometry was performed to measure cTnI concentration by immersion of the electrodes into the wells. Based on prior characterization, micro-MWCNT electrodes were selected for evaluation of the cTnI assay. Figure 4A shows the generic increasing trend between cTnI concentration and current in diluted serum samples. In Figure 4B, the undiluted serum samples have greater variability, particularly at higher concentrations and a reduced signal magnitude. Figure 4C shows the undiluted serum dose response, analogous to the optical assay, achieving an improved LOD of <3.125 pg mL^–1^ in 10% spiked serum. In Figure 4D, the LOD corresponding to the undiluted serum samples assay was 7.4 pgmL^–1^. While the overall signal magnitude was reduced compared to diluted serum, improved differentiation between lower concentrations was maintained. The highest cTnI standard produced ∼5 nA in 10% serum and <3 nA in undiluted serum, while background currents remained similar, resulting in an approximately two-fold increase in the calculated LOD. Nevertheless, the electrodes maintained sub-10 pg mL^–1^ sensitivity in undiluted serum, highlighting the robustness of these low-cost, in-house fabricated devices. The reduced signal magnitude and increased variability observed in undiluted serum are attributed to matrix effects associated with the high protein content and endogenous electroactive species in whole serum, which can partially hinder electron transfer and substrate diffusion in HRP-labeled electrochemical sensors. These effects increase background contributions and signal dispersion, particularly at higher analyte concentrations, while preserving the overall concentration-dependent trend. The observed nonlinear amperometric response is consistent with previous reports and is attributed to surface effects, binding equilibria, mass transport limitations, and site saturation.^28,42^ In contrast, macro-MWCNT electrodes exhibited increased variability and a poorer LOD of 140 pg mL^–1^ in 10% serum (Figure S1.7), indicating electrode size and the associated mass-transport regime strongly influence analytical sensitivity. In Figure 4C,D standard deviation of the steady-state current increased at higher cTnI concentrations consistent with partial saturation of the immunoassay and enzymatic signal generation. At elevated enzyme loading, rapid accumulation of oxidized TMB can lead to signal compression and increased variability, a behavior commonly reported for enzyme-amplified electrochemical assays. Importantly, this effect occurs beyond the clinically relevant concentration range and does not compromise the assay sensitivity or precision in the low-concentration regime used for diagnosis.

**Figure 4:**
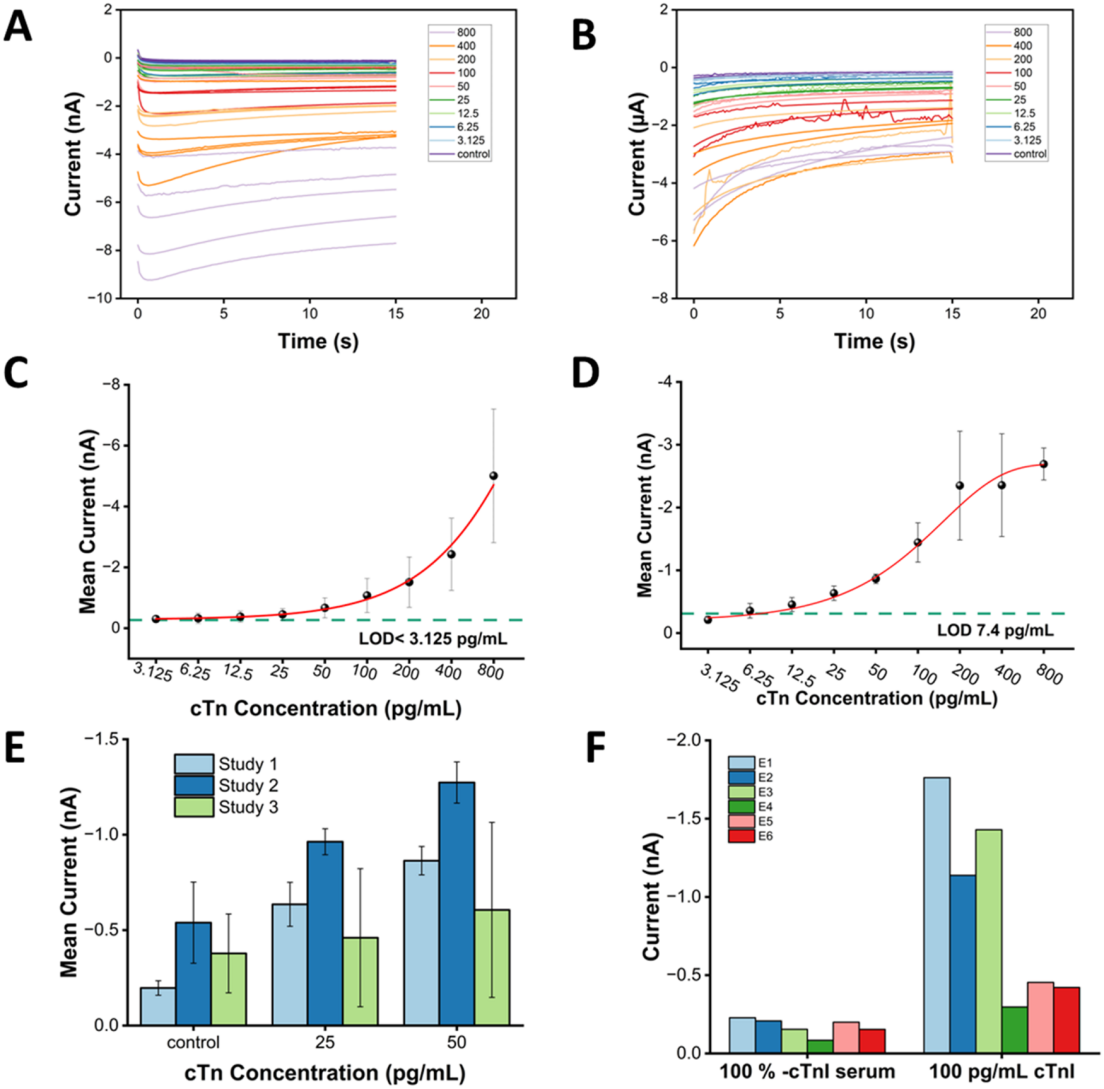
Amperometric characterization of the electrochemical immunoassay using micro-MWCNT electrodes. (A) Chronoamperometric current response over 15 s for assays performed in diluted (10%) human serum. (B) Chronoamperometric current response for the same assay performed in undiluted human serum. (C) Dose–response curve for cTnI spiked into 10% human serum, showing mean steady-state current ± 1× SD (*n* = 5). The teal dashed line indicates the limit of detection (LOD). (D) Dose–response curve for cTnI spiked into undiluted human serum, showing mean steady-state current ± 1× SD (*n* = 3). The teal dashed line indicates the LOD. (E) Reproducibility of the assay across three nonconsecutive days (*n* = 4 per study; four electrodes used throughout), comparing mean chronoamperometric responses for nonspiked and spiked cTnI in undiluted human serum. (F) Specificity assessment using six micro-MWCNT electrodes (E1-E6), comparing current responses to cTnI-depleted serum (negative control) and serum spiked with 100 pg mL^–1^ cTnI (*n* = 6).

Wash steps performed to remove excess reagents from wells before and are intrinsic to immunoassay operation and do not reduce the analytical challenge imposed by the sample matrix. The critical antigen–antibody recognition event occurs entirely in undiluted human serum, such that sensor performance is evaluated under highly complex and clinically relevant conditions despite subsequent standardized wash steps. While further optimization and standardization will be required to meet clinical validation standards, the current performance establishes a strong foundation for continued development toward translational applications.

Next, the reproducibility and selectivity were examined. Figure 4E shows the mean current obtained from three independent assays. Studies 1 and 2 exhibited comparable mean currents with minor differences attributed to electrode-to-electrode variability. On Day 1 and Day 2, a clear separation between the control and both spike levels was observed, as indicated by nonoverlapping error bars. In contrast, Day 3 exhibited increased variability across all samples, reflected by larger standard deviations rather than a systematic shift in the mean signal. This suggests global assay instability, likely arising from reagent degradation over time, rather than concentration-dependent effects, indicating a need for improved reagent stabilization. Additional variability may arise from the nonuniform distribution of conductive materials across individual electrode surfaces, influencing electrochemical behavior.

Antibody specificity was maintained throughout, with a minimal background signal observed in both optical and electrochemical measurements. Assays were performed in high-quality cTnI-depleted pooled human serum, providing a clinically relevant and challenging matrix containing endogenous proteins, electrolytes, and metabolites that can affect electron transfer and binding behavior. As shown in Figure 4F, the negative control remained consistent across multiple samples (*n* = 6), indicating a low baseline signal and confirming that the measured response originates from specific antigen–antibody interactions rather than nonspecific adsorption. Collectively, these results demonstrate acceptable selectivity in complex biological matrices suitable for proof-of-concept evaluation, with detailed cross-reactivity studies reserved for future validation.

The sensitivity achieved here outperformed previous work, which used thin gold film electrodes and measured cTnI concentration indirectly via TMB^+^ detection without surface functionalization. As summarized in Table 1, both macro- and micro-PLA/MWCNT electrodes provided substantially lower LODs under comparable conditions, with electrode miniaturization yielding a further marked improvement. The attainment of sub-5 pg mL^–1^ sensitivity using low-cost, additively manufactured composite electrodes underscores the importance of electrode geometry and electrocatalytic activity in influencing analytical performance.

**Table 1:** Electrochemical Immunoassays with Off-Electrode Target Capture and Final Electrochemical Readout

Electrode Material	Serum Content	LOD (pg mL^–1^)	Reference
Thin gold film electrode	10%	1000	^7^
macro-MWCNT	10%	140	This work
micro-MWCNT	10%	<3.125	This work
micro-MWCNT	100%	7.4	This work

Due to the several advantages of the 3DP PLA/MWCNT electrodes, i.e., their straightforward, sustainable, and inexpensive fabrication, and the ability to customize geometries to suit application needs, their use is justified. The next step was to reduce the time to result while maintaining adequate sensitivity and reproducibility. To this end, the duration of assay incubation steps was shortened and the impact of incubation temperature and sample mixing was investigated.

### Optimization of Immunoassay Conditions to Reduce Assay Time

To improve clinical relevance, incubation times were reduced to ∼15 min, and the effects of temperature and agitation were evaluated to identify optimal conditions. The new conditions were first tested using optical screening to construct a dose response curve, calculate SNR and LODs for comparison of incubation conditions (Figure 5A–C).

**Figure 5:**
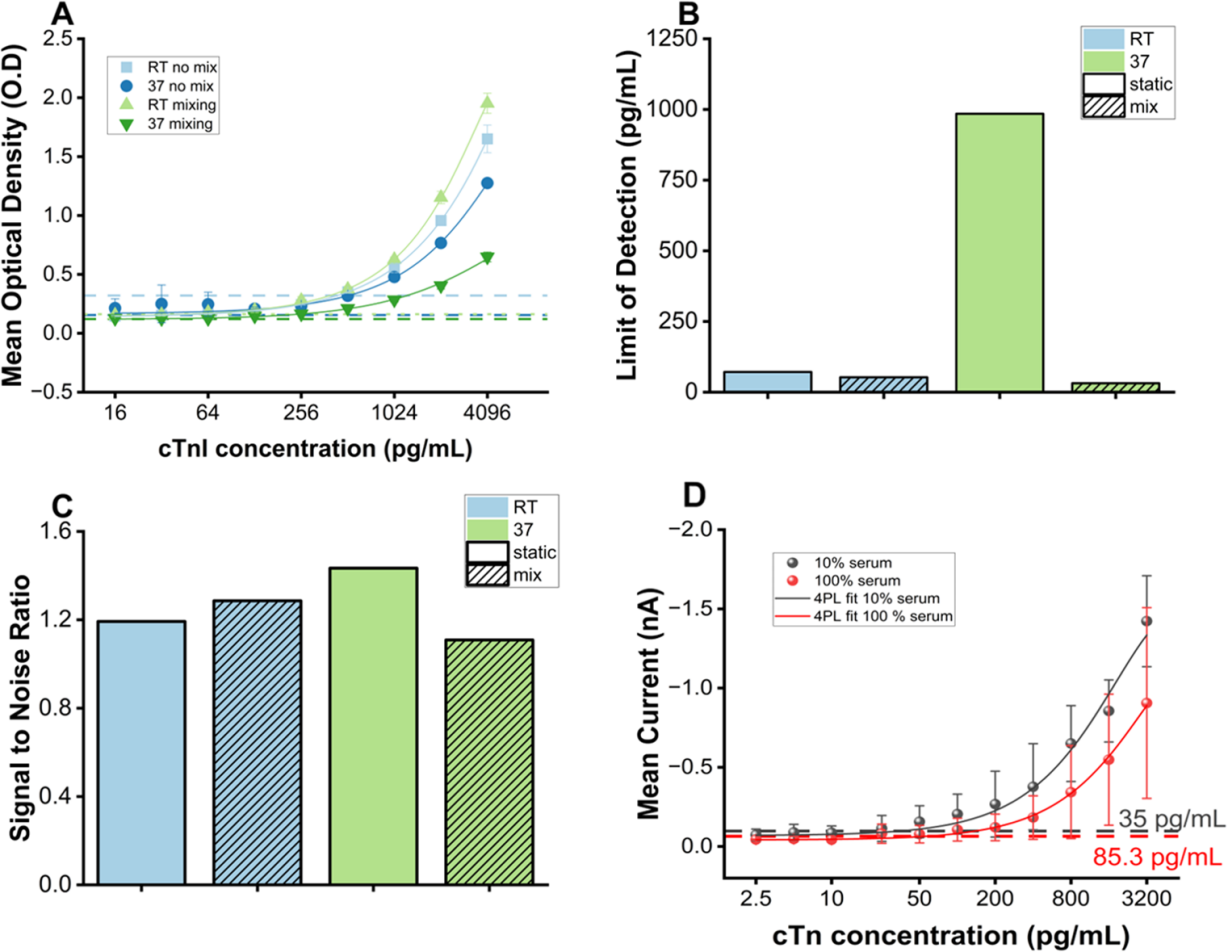
Fast absorption-based immunoassay screening and electrochemical validation. Screening of different conditions at room temperature without mixing, at room temperature with mixing, at 37 °C without mixing, and at 37 °C with mixing in 10% cTnI-depleted serum. (A) Dose–response curves for each condition, shown as mean optical density (OD) ± 1× SD (*n* = 3); dashed lines indicate condition-specific LODs. (C) Signal-to-noise ratios (SNRs) using the negative control and 100 pg mL^–1^ standard in 10% serum (log 10 *y*-axis). For panels B and C, the *Y*-axis is displayed on a logarithmic scale to visualize differences across orders of magnitude. (D) Electrochemical validation of optimised incubation conditions by measuring mean steady-state current ± 1× SD using 10% human serum (gray) and undiluted human serum (red), with LODs displayed as dashed lines (*n* = 4).

Figure 5A shows that mixing during incubation at 37 °C resulted in a decreased optical signal and background noise. Figure 5B shows that at room temperature, static and mixed incubations produced moderate SNRs of 1.33 and 1.98, with corresponding LODs of 71.5 pg mL^–1^ and 52.8 pg mL^–1^. Figure 5C indicates that at 37 °C, static incubation decreased SNR to 0.86 but resulted in a much higher LOD of 984.7 pg mL^–1^, indicating greater background interference. In contrast, mixed incubation at 37 °C yielded a lower SNR of 3.22 and the lowest LOD of 31.6 pg mL^–1^, demonstrating improved sensitivity and more reproducible detection. The results summarized in Table 2 indicate both temperature and agitation influence assay performance, with mixing at 37 °C providing the most favorable LOD of the shorter incubation conditions. Compared to the 2 h incubation, the accelerated immunoassay had a greater SNR and a LOD (<3.125 pg mL^–1^ vs 31.6 pg mL^–1^).

**Table 2:** Optical Absorbance-Based Assays Performed in This Work^a^

Incubation time	Incubation temperature	Incubation mixing	SNR	LOD (pg mL^–1^)
2 h	room	static	2.35	<3125
15 min	RT	static	1.33	71.5
15 min	RT	mix	1.98	52.8
15 min	37	static	0.86	984.7
15 min	37	mix	3.227	31.6

a The table summarizes the results presented in Figure 5A–C. All assays used 10% cTnI-depleted serum.

Figure 5D illustrates the electrochemical dose response obtained using the fast immunoassay protocol. Lower currents were generated for positive samples compared with the longer immunoassay protocol (Figure 4C). The electrochemical LOD in 10% serum was 35 pg mL^–1^, comparable to the fast optical measurements under the same conditions (∼31.6 pg mL^–1^). For the undiluted serum cTnI test, the overall signal decreased, resulting in a calculated LOD of 85.2 pg mL^–1^. These observations suggest that while the fast protocol enables rapid detection, further optimization is needed to completely mitigate matrix interference and recover maximal sensitivity in complex samples. A cTnI concentration of ∼85 pg mL^–1^ lies above the established 99th-percentile upper reference limits for high-sensitivity cardiac troponin assays and therefore falls within the clinically relevant range associated with myocardial injury in acute myocardial infarction.

Reports show 3DPEs can be applied as immunosensors when combined with nanomaterial surface modification, with several studies showing a low pg mL^–1^ range for viral proteins and other biomarkers.^17,42,43^ Many reports use viral pathogens to achieve strong performance, because viral targets are arguably easier to detect due to their size and multivalent binding.^44^ A recent study employed novel 3D-printed interdigitated electrodes for cTnI troponin detection, reporting an LOD of 76.96 pg mL^–1^. However, the sample matrix used to determine this LOD was not specified, and the lowest concentration tested in real sample recovery experiments was 1 ng.^45^ Otherwise, minimal work has been done showcasing 3DPEs and the quantification of cardiac troponin in human serum.

The HRP/TMB reporter system was deliberately selected to build upon our previous work, allowing direct comparison with established electrochemical platforms.^46^ Table 1 compares the LOD for off-electrode antigen capture highlighting the substantial improvement from previously reported off-electrode cTnI detection limits of 1000 pg mL^–1^. Previous work also developed a chemisorption based cTnI electrochemical immunosensor an LOD of 109 pg mL^–1^ in 10% serum.^7^ In contrast, our platform demonstrates improved sensitivity, reaching 7.4 pg mL^–1^ under similar incubation conditions and 85 pg mL^–1^ in undiluted serum in a shorter time frame, demonstrating the performance benefits of the novel micro-MWCNT electrodes. With further work, sensitivity could be improved to reach the clinically relevant 15 pg mL^–1^ while maintaining shorter incubations for a faster sample-to-result time. This significant improvement highlights the substantial impact of the MWCNT-based electrode architecture. Moreover, the system is cost-effective, with each electrode costing only 13 pence to manufacture and utilizing straightforward chronoamperometry.

The Consistent Dipper shortened sample analysis time and improved consistency across samples. In comparison, commercial platforms such as Legion arrays^47^ and DropSens screen-printed 96-well plates^48^ enable high-throughput electrochemical analysis but have notable limitations. Legion provides fully parallel measurements yet requires complex instrumentation, while DropSens screen-printed plates are extremely costly, single-use, and have fixed geometries. In contrast, reported 3D-printed electrochemical cells presented previously in the literature highlight the low cost and design flexibility of additive manufacturing, yet these devices are typically standalone housings or flow cells that lack microplate compatibility. A recent study fixed CB/PLA electrodes directly into microplates removes the need for support structures but requires direct biomolecule immobilization.^49^ The Consistent Dipper addresses these gaps by combining low-cost, in-house fabrication with direct 96-well integration and a modular TED adaptable to various electrode types, facilitating reproducible electrochemical testing.

It is worth noting that HRP/TMB-based immunoassays are constrained by enzyme stability, variability, and the ∼30 min incubation required for sufficient TMB oxidation. There are examples of ultrasensitive enzymatic-based cTnI sensors, but they typically employ more complex fabrication methods.^10^ Alternatively, nonenzymatic approaches can offer faster and more sensitive alternatives, often bypassing multistep substrate incubations, and platforms such as microfluidic electrochemical arrays^50^ or one-step digital immunoassays^51^ have demonstrated clinically relevant turnaround times of 10–15 min with very low LODs. Future work will involve testing patient-derived samples to validate assay robustness and could explore nonenzymatic approaches to develop a user-friendly, sensitive, and rapid test.

## Conclusions

This study demonstrated that PLA/MWCNT microelectrodes are capable of high-sensitivity cTnI electrochemical detection in pooled serum. This work is believed to be the first to demonstrate the use of in-house-produced 3D-printed microelectrodes for biological measurements of complex samples. Electrode material screening revealed that the incorporation of MWCNTs enhanced electron transfer efficiency compared to carbon black filaments, and reducing the electrode size to ∼ 0.1 mm elicited a microelectrode-like response. The system utilizes highly specific antibodies to capture cTnI and an HRP/TMB-based label system, paired with microsized PLA/MWCNT electrodes to attain exceptional sensitivity without the need for electrode functionalization or modification. The Consistent Dipper facilitated rapid electrode transfer between wells, shortening acquisition time and improving assay throughput. By minimizing movement-induced artifacts, it also lowered noise and measurement variability, contributing to enhanced analytical sensitivity. This configuration achieved an impressive LOD below 10 pg mL^–1^ for cTnI in undiluted serum. Accelerated incubation conditions showed promise with an LOD of 85 pg mL^–1^. Moving forward, the platform will be translated to other diagnostic markers, and signal-amplification strategies can be examined to further improve sensitivity, followed by more extensive system integration.

## Supplementary Material







## Notes

Details further imaging and schematics of the electrodes and the Consistent Dipper (Supporting 2) (PDF)
Details the reagents list and Supporting results figures (Supporting Information 1) (PDF)
Use of the Consistent Dipper is also available (Video S1) (MP4)
