# Supplementary material for: Development of a High-Sensitivity Electrochemical Immunoassay Using a Fully 3D-Printed Electrocatalytic Microelectrode Probe Platform
Source: Anal Chem. 2026 Mar 19;98(12):9260–72. doi: 10.1021/acs.analchem.5c08037 (PMC13044883; doi:10.1021/acs.analchem.5c08037)
Supplement: Supplementary file 1 [file ac5c08037_si_001.pdf]

# Supplementary information 2

# Development of a high-sensitivity electrochemical immunoassay using a fully 3D-printed electrocatalytic microelectrode probe platform.


Niamh Docherty[1], Chloe L Miller[2], Alexandra Dobrea[1,3], Daniel Macdonald[1], Alisdair Gordon[1,3], Susan Pang[4], Ying Fu[5], Melanie Jimenez[3*], Damion K Corrigan[1*] and Bhavik Anil Patel[2*]

1. University of Strathclyde, Centre for Advanced Measurement Science and Health Translation, Pure and Applied Chemistry, Thomas Graham Building, 295 Cathedral St, Glasgow G1 1XL, UK.
2. School of Applied Sciences, Centre for Lifelong Health, University of Brighton, Brighton, East Sussex BN2 4GJ, U.K.
3. University of Strathclyde, Biomedical Engineering, Wolfson Centre, 106 Rottenrow East, G4 0NW Glasgow
4. National Measurement Laboratory at LGC, The Priestley Building, Guildford, Surrey, GU2 7XY
5. University of Strathclyde, Pure and Applied Chemistry, Technology Innovation Centre, 99 George Street, Glasgow G1 1RD,

*melanie.Jimenez@strath.ac.uk , damion.corrigan@strath.ac.uk, b.a.patel@brighton.ac.uk



Figure S2. 1: Photograph of a fresh micro MWCNT electrode (A) and a prepared electrode (B)..........2
Figure S2. 2: Photograph of The Consistent Dipper components ..........................................................2
Figure S2. 3: The electrode holder for the Consistent Dipper set up......................................................3
Figure S2. 4: Isometric View of the Consistent Dipper...........................................................................3
Figure S2. 5: Exploded View of the Consistent Dipper. ........................................................................4

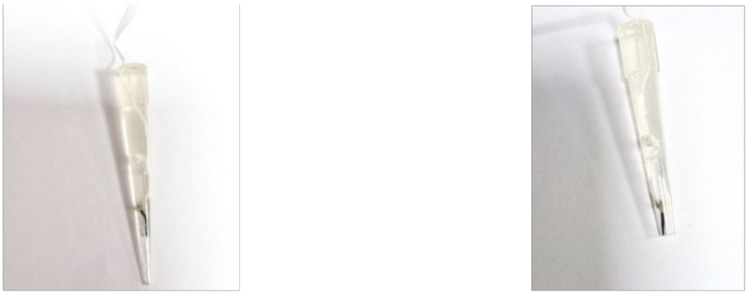


*Figure S2. 1: Photographs of a fresh micro MWCNT electrode and a prepared electrode.*

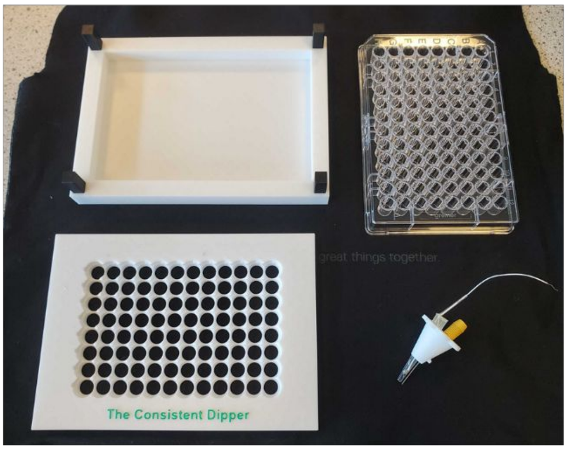


*Figure S2. 2: Photograph of The Consistent Dipper components*

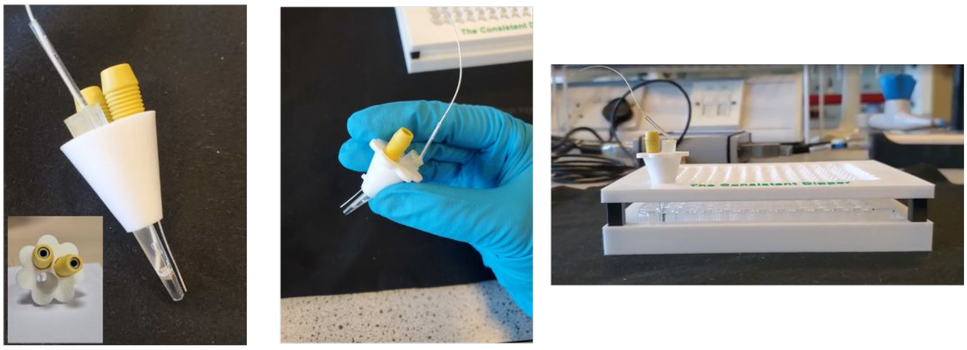

**2 mm banana sockets interface directly with the potentiostat cables**

*Figure S2. 3: The electrode holder for the Consistent Dipper set up.*

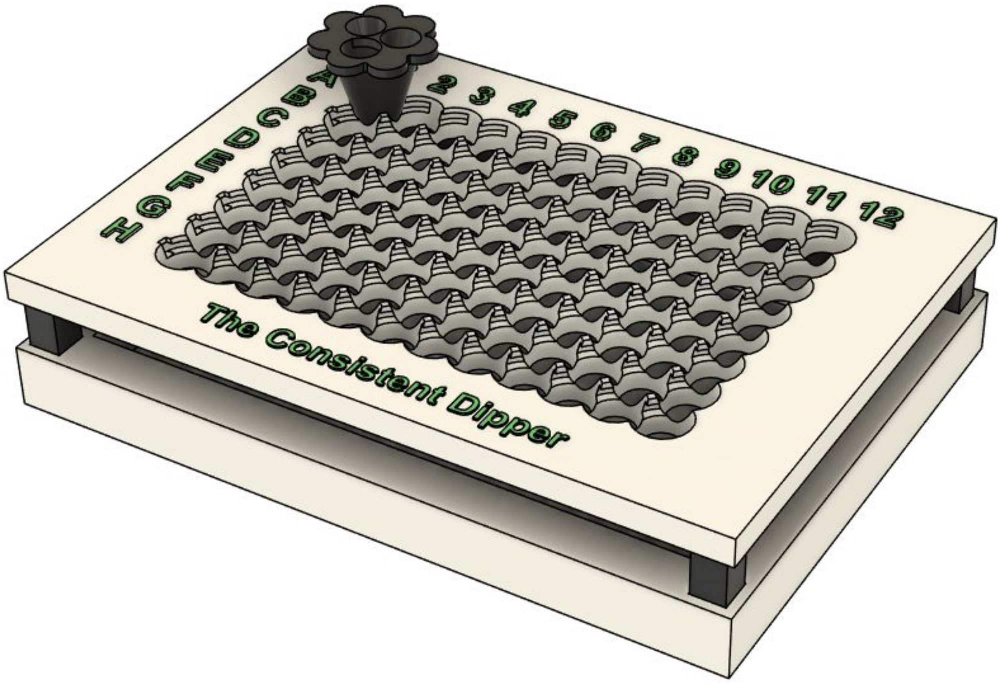


*Figure S2. 4: Isometric View of the Consistent Dipper*

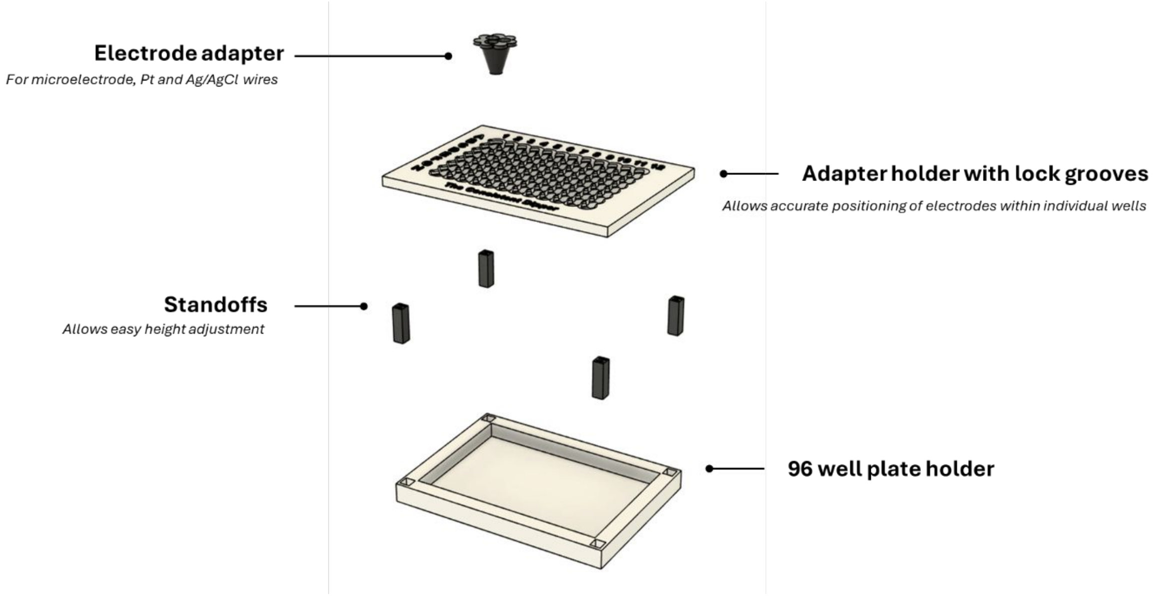


*Figure S2. 5: Exploded View of the Consistent Dipper.*
