# Supplementary material for: Development of a High-Sensitivity Electrochemical Immunoassay Using a Fully 3D-Printed Electrocatalytic Microelectrode Probe Platform
Source: Anal Chem. 2026 Mar 19;98(12):9260–72. doi: 10.1021/acs.analchem.5c08037 (PMC13044883; doi:10.1021/acs.analchem.5c08037)
Supplement: Supplementary file 2 [file ac5c08037_si_002.pdf]

Supplementary information 1

Development of a high-sensitivity electrochemical immunoassay using a fully 3D-printed electrocatalytic microelectrode probe platform.

Niamh Docherty[1], Chloe L Miller[2], Alexandra Dobrea[1,3], Daniel Macdonald[1], Alisdair Gordon[1,3], Susan Pang[4], Ying Fu[5], Melanie Jimenez[3*], Damion K Corrigan[1*] and Bhavik Anil Patel[2*]

1. University of Strathclyde, Centre for Advanced Measurement Science and Health Translation, Pure and Applied Chemistry, Thomas Graham Building, 295 Cathedral St, Glasgow G1 1XL, UK.

2. School of Applied Sciences, Centre for Lifelong Health, University of Brighton, Brighton, East Sussex BN2 4GJ, U.K.

3. University of Strathclyde, Biomedical Engineering, Wolfson Centre, 106 Rottenrow East, G4 0NW Glasgow

4. National Measurement Laboratory at LGC, The Priestley Building, Guildford, Surrey, GU2 7XY

5. University of Strathclyde, Pure and Applied Chemistry, Technology Innovation Centre, 99 George Street, Glasgow G1 1RD,

*melanie.Jimenez@strath.ac.uk , damion.corrigan@strath.ac.uk, b.a.patel@brighton.ac.uk

Table of Contents

Antibodies, reagents and buffer solutions. ……………………………………………….....2
Figure S1.1: Characterisation of 3D printed electrodes Cyclic voltammogram....................................2
Figure S1. 2: *RAMAN spectroscopy of the micro-CNT electrode.* 3
Figure S1. 3 Effect of 0.5 M NaOH multistep amperometry pretreatment (n=3). 3
Figure S1. 5: The original electrochemical set-up...4
Figure S1. 6: Optical density data of the cTnI Immunoassay...5
Figure S1. 7: Amperometric data generated using macro-MWCNT electrodes....................................6

Antibodies, reagents and buffer solutions.

Troponin I Type 3 (cardiac) antibody (clone 19C7cc, BSA-free, Cat. No. NB110-2546) and HRP-conjugated Troponin I Type 3 (cardiac) antibody (clone 4C2cc, Cat. No. NB110-8453H) were purchased from Novus Biologicals. Human cardiac troponin I (cTnI)-free serum (Cat. No. 8TFS2) and recombinant human cardiac troponin IC complex (Cat. No. 8ICR3) were obtained from HyTest. Sodium carbonate and sodium bicarbonate (analytical grade, Sigma-Aldrich/Merck) were used to prepare 50 mM buffer solutions at pH 9.4. Bovine serum albumin (BSA, Sigma-Aldrich/Merck) was used to prepare 1 % BSA solutions in PBS. Phosphate-buffered saline (PBS, 1×, pH 7.4) was prepared with or without additives, including 0.05 % Tween-20 (PBS-T) or 1 % BSA (PBS-BSA), using analytical grade reagents (Sigma-Aldrich/Merck).

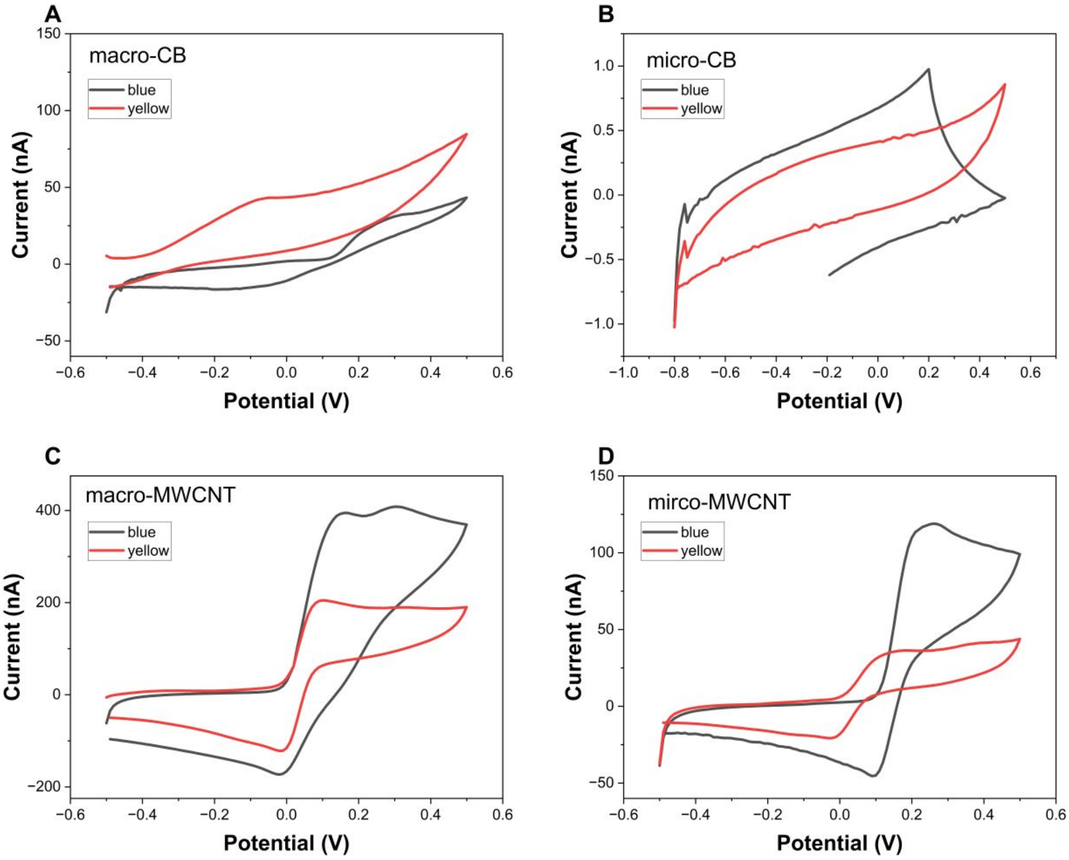

Figure S1.1: Cyclic voltammetric characterisation of 3D-printed electrodes

A) The redox states of TMB. CVs were performed in both TMB^{+} ELISA product solution (blue) and TMB^{2+} solution (yellow) for each electrode type; macro-CB, micro-CB, macro-MWCNT, micro-MWCNT electrodes in TMB substrate- HRP solution

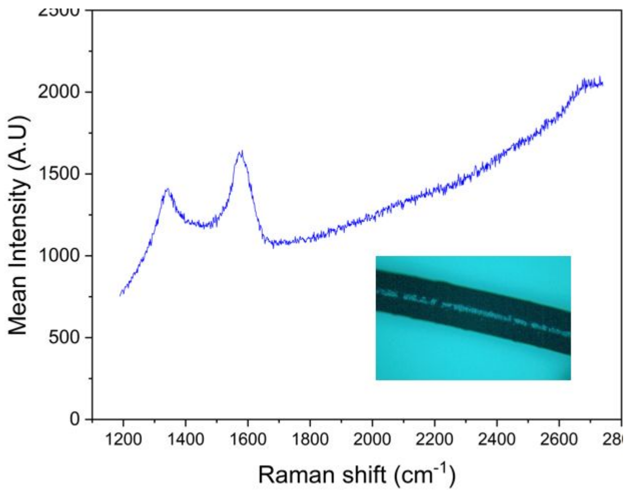

Figure S1. 2: *RAMAN spectroscopy of the micro-CNT electrode.*

Raman spectra were collected with a 532 nm laser excitation (maximum power output 50 mW), 1800 lines/mm grating, 20 x objective and an integration time of 5 seconds. White light image was taken using a 5x objective.

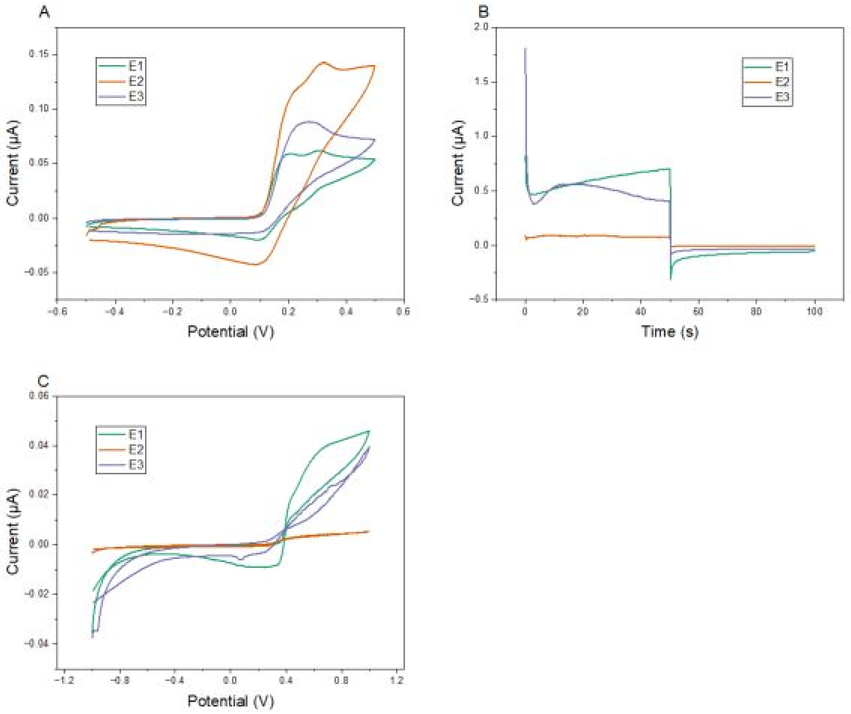

Figure S1. 3 Effect of 0.5 M NaOH multistep amperometry pretreatment (n=3).

A). Cyclic voltammetry (-0.5 to 0.5 V, 0.1 V/s) of TMB substrate solution (TMB:H_2O_2) using freshly sanded micron-sized CNT electrodes. B) Alkaline pretreatment via multistep amperometry (50 s at 1.4 V followed by 50 s at -1.0 V). C) Repetition of the TMB substrate solution cyclic voltammetry to compare the effect of the NaOH pretreatment.

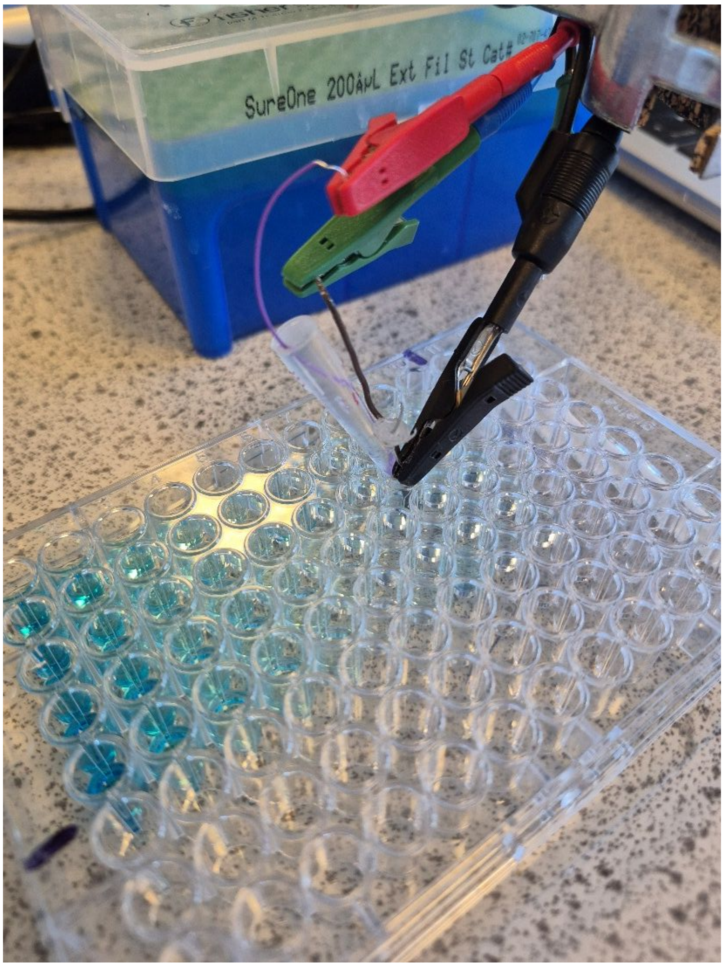

Figure S1. 4: The original electrochemical set-up.

The 3D printed working electrode (red clip) with a bleached silver wire reference electrode (green) and a platinum wire (black) electrode. The electrodes were stabilised with a pipette box or clamp stand. Moving each electrode between wells was laborious for larger experiments.

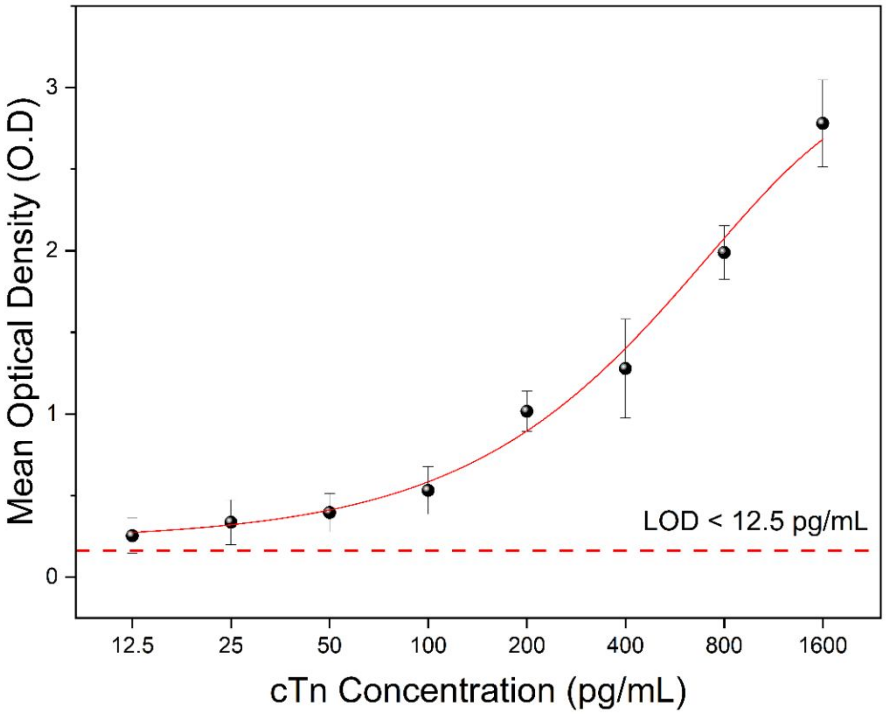

Figure S1. 5: Optical *density data of the cTnI Immunoassay.*

Dose response curve of mean optical density (O.D) against cTnI concentration (pg mL $^{-1}$). LOD depicted using red dashed line. Error bars 1x STD.

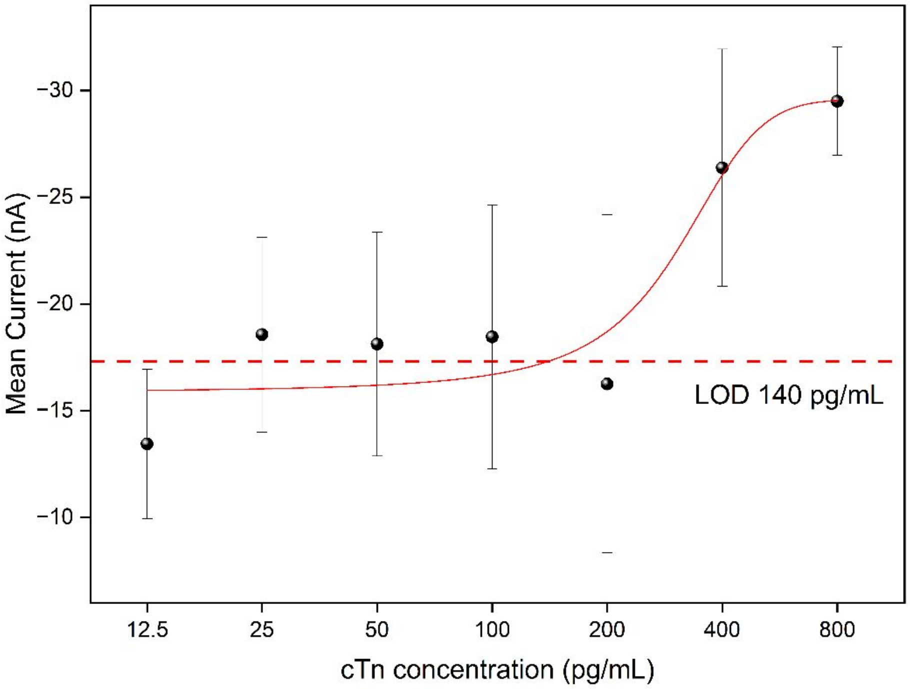

Figure S1. 6: Amperometric *data generated using macro-MWCNT electrodes.*

Dose response curve of spiked cTnI (pg mL $^{-1}$) in 10 % human serum (n=3) and mean current (nA)). 4Pl fit shown by red line. Error bars are 1x STD
